# Subjective Age Changes During the COVID-19 Pandemic

**DOI:** 10.1093/geroni/igab046.2270

**Published:** 2021-12-17

**Authors:** Antonio Terracciano

**Affiliations:** Florida State University, Florida, United States

## Abstract

Aging is associated with an increased risk of COVID-19 morbidity and mortality. In this study, we tested whether the pandemic influenced how old individuals felt by examining longitudinal within-person changes in subjective age. We tested two alternative hypotheses: (a) people felt increasingly older in response to the stress generated by COVID-19; (b) people felt increasingly younger due to psychological distancing from older age. We tested these hypotheses in a large US sample of adults assessed once before and twice during the COVID-19 pandemic. Multilevel analyses indicated that people reported feeling younger with the emergence of COVID-19. We further tested demographic, health, and psychosocial predictors of changes in subjective age. Overall, the findings supported the hypothesis that subjective age partly reflects a coping process of psychological distancing from older age, a process that parallels physical and social distancing.

